# Prediction of ambulatory outcome in patients with corona radiata infarction using deep learning

**DOI:** 10.1038/s41598-021-87176-0

**Published:** 2021-04-12

**Authors:** Jeoung Kun Kim, Yoo Jin Choo, Hyunkwang Shin, Gyu Sang Choi, Min Cheol Chang

**Affiliations:** 1 Department of Business Administration, School of Business, Yeungnam University, Gyeongsan-si, Republic of Korea; 2 Department of Rehabilitation Medicine, College of Medicine, Yeungnam University, 317-1, Daemyungdong, Namku, Taegu, 705-717 Republic of Korea; 3 Department of Information and Communication Engineering, Yeungnam University, Gyeongsan-si, Republic of Korea

**Keywords:** Neuroscience, Diseases, Neurology

## Abstract

Deep learning (DL) is an advanced machine learning approach used in diverse areas such as bioinformatics, image analysis, and natural language processing. Here, using brain magnetic resonance imaging (MRI) data obtained at early stages of infarcts, we attempted to develop a convolutional neural network (CNN) to predict the ambulatory outcome of corona radiata infarction at six months after onset. We retrospectively recruited 221 patients with corona radiata infarcts. A favorable outcome of ambulatory function was defined as a functional ambulation category (FAC) score of ≥ 4 (able to walk without a guardian’s assistance), and a poor outcome of ambulatory function was defined as an FAC score of < 4. We used a CNN algorithm. Of the included subjects, 69.7% (n = 154) were assigned randomly to the training set and the remaining 30.3% (n = 67) were assigned to the validation set to measure the model performance. The area under the curve was 0.751 (95% CI 0.649–0.852) for the prediction of ambulatory function with the validation dataset using the CNN model. We demonstrated that a CNN model trained using brain MRIs captured at an early stage after corona radiata infarction could be helpful in predicting long-term ambulatory outcomes.

## Introduction

Stroke is a leading cause of disability in humans, and ambulatory dysfunction is one of the most severe disabling sequelae^1^. For stroke patients, the accurate prediction of ambulatory dysfunction is crucial in terms of rehabilitation. Several methods have been proposed to estimate the ambulatory outcomes in stroke patients based on clinical findings, imaging studies [magnetic resonance imaging (MRI) and computed tomography (CT)], and electrophysiological studies^2–5^. Recently, various methods such as diffusion tensor imaging and functional magnetic resonance have been employed to increase the motor-function prediction accuracy^6,7^.

Machine learning (ML) entails computer algorithms that can automatically learn from data without requiring explicit programming^8^. Recently, ML techniques have been applied to diverse areas such as bioinformatics, image analysis, and natural language processing^9^. The use of ML helps overcome the limitations of existing techniques and has enabled breakthroughs in several areas. Some previous studies used ML for predicting motor function after a stroke with clinical data as input variables^10–12^. However, an excessively large number of input variables were used; moreover, the clinical data obtained in various hospitals were different. Therefore, it was not possible to use an ML algorithm designed for one hospital in another hospital. Imaging studies, such as brain MRI and CT, could performed for all stroke patients in all clinics. Consequently, an algorithm that employs brain imaging results as input variables can be widely used.

The deep learning (DL) technique is one of the advanced ML approaches. Particularly, it constructs artificial neural networks with structures and functions similar to those of the human brain by using a large number of hidden layers^13^. The DL technique can outperform traditional ML techniques and can learn from unstructured and perceptual data such as images and languages. A convolutional neural network (CNN) is a representative DL model that has a significant advantage in imaging recognition and classification^14^.

In this study, using brain MRI data obtained at early stages of corona radiata infarcts, we developed a CNN to predict the ambulatory outcome of corona radiata infarction at six months after onset.

## Results

The receiver operating characteristic curve analysis and AUC calculation were performed using scikit-learn. Of the 221 patients, 101 (45.7%) showed a favorable outcome of ambulatory function at the six-month follow-up after stroke onset and 120 (54.3%) showed a poor outcome.

**Figure 1:**
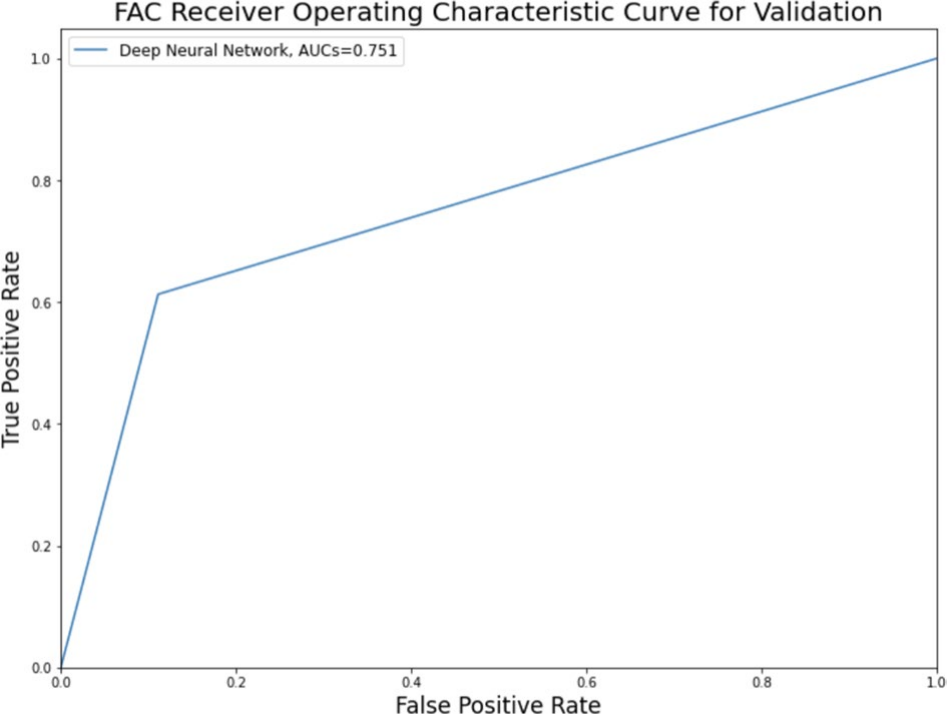
Functional ambulation category (FAC) receiver operating characteristic curve and area under the curve for data validation.

In the prediction of ambulatory function with the validation dataset using the CNN model, the AUC was 0.751 (95% CI 0.649–0.852) (Fig. 1).

## Discussion

In the present study, we developed a CNN model for predicting the ambulatory outcome six months after corona radiata infarction using MR images as input data.

In our study, the AUC of the model, evaluated with the validation dataset, was 0.751 with regard to predicting the ambulatory function at six months after the onset of corona radiata infarction. Considering that AUCs ranging from 0.7 to 0.8 are generally considered acceptable^15^, the proposed CNN model trained using brain MRI input data obtained at an early stage of cerebral infarction can be helpful for clinicians in predicting long-term ambulatory outcomes.

A deep neural network (DNN) is characterized by a multilayer perceptron with multiple hidden layers or a feedforward neural network, which provides greater ability than a traditional shallow neural network^13^. A CNN is representative of a DNN model; it receives multiple channels of two-dimensional data as input and transforms them repeatedly using convolution and pooling operations^14^. These processes allow the extraction of valuable features from the input data. CNNs are widely used in image processing and pattern recognition. In prior reports on the prediction of the motor outcome of cerebral infarction using images, infarct volume and leukoaraiosis were reported to be correlated with post-infarction motor outcomes^16,17^. Apart from these factors, damage to the corticospinal tract (CST) is also known to be a decisive factor for motor prognosis. Therefore, for the accurate prediction of motor outcomes, the state of the CST should also be evaluated. To evaluate the state of the CST, diffusion tensor tractography is used^7^. However, this method is limited because of false negatives and positives^18^. We proposed that a CNN model could determine whether cerebral infarcts involved areas through which the CST passed. In addition, during the development of the algorithm, we determined that the infarct volume and degree of leukoaraiosis should be considered for predicting the motor outcome. However, owing to the nature of the DNN, we could not know which factors the algorithm considered (and the manner in which they were weighted) for predicting the ambulatory outcome after corona radiata infarction.

To date, several studies have evaluated the application of machine learning to predict the motor outcome after a stroke^10–12^. Heo et al. predicted the modified Rankin Scale score at 3 months after ischemic stroke using deep neural network, logistic regression, and random forest with 2604 acute ischemic stroke subjects. They used 38 variables including patient demographics, initial National Institutes of Health Stroke Scale scores, stoke subtypes, and time from onset to admission as inputs. The AUCs were 0.888, 0.849, and 0.857, respectively^10^. Sale et al. studied the predictability of improving motor function after rehabilitation treatment from the early stages of stroke. They used 55 patients' data collected at the time of admission to the Department of Rehabilitation Medicine and discharge, and predicted the Barthel Index and functional independence measure score with a linear support vector machine regression model. All output results and the actual measured results showed a good correlation of 0.75–0.81^12^. Lin et al. constructed a prognostic model of functional outcome using data from 313 patients with stroke. Various functional measurement outcomes at early stage after storke, such as modified Rankin Scale and Barthel Index scores, gait speed, and results of Mini-Mental State Examination, were used as inputs. The Barthel index status at discharge was predicted. They utilized logistic regression, support vector machine, and random forest models, and AUCs were 0.792, 0.774, and 0.792, respectively^11^. Although the AUCs of the previous studies tend to be higher than that of our study, they used clinical data as input variables for predicting the motor prognosis, without using image data. For developing algorithms using clinical data, an excessively large number of variables are necessary. In addition, each hospital collects different types of clinical data. Therefore, in clinical practice, it would be difficult to globally use a specific model developed using clinical data as input variables. In contrast to previous models, we used only MR images to develop the model. Brain MRI is used for diagnosing cerebral infarction in every hospital; therefore, we believe that a DL algorithm developed using brain MR images can be practically used in clinical practice. To the best of our knowledge, our study is the first to show the possibility of using DL algorithms trained using MR images for predicting the ambulatory outcomes after a stroke.

Our study was limited in that we used a small number of brain MRI data for training the CNN model. We think that, if a larger number of input MRI data are used, the accuracy of the model will increase. In addition, we believe that developing a model that integrates MR images and patients’ clinical data would be helpful for increasing the accuracy of the model for predicting the ambulatory outcomes after corona radiata infarction.

In conclusion, we demonstrated that a CNN model trained using brain MRIs captured at an early stage after corona radiata infarction could be helpful for predicting long-term ambulatory outcomes. The accuracy of the model was acceptable, but not considerably high. Therefore, it should be used as a supplementary tool for clinicians to predict long-term ambulatory outcomes. In addition, we believe that the combined use of clinical data with brain MRI as input data may be helpful for increasing the accuracy of the model for predicting the ambulatory outcomes after corona radiata infarction.

## Materials and methods

### Subjects

We retrospectively recruited 221 consecutive patients with corona radiata infarcts (mean age = 65.0 ± 11.9, M:F = 115:106) who underwent stroke rehabilitation in a single university hospital from January 2003 to January 2020 for this study (Table 1). The inclusion criteria were as follows: (1) first-ever stroke; (2) age over 20 years; (3) hemiplegia or hemiparesis following corona radiata infarction; (4) brain MRI conducted within 30 days after onset; (4) functional ambulatory category (FAC) checked six months after stroke onset; and (5) absence of other serious medical complications, such as pneumonia or cardiac problems, during the period from onset to final evaluation. The study protocol was approved by the institutional research board of Yeungnam university hospital (No. 2019-10-008). The institutional research board waived the need for informed consent for this study since we used de-identified retrospective data. This study followed Helsinki Declarations.

**Table 1:** Patient demographic and clinical data collected on the day of transfer or admission to the rehabilitation department.

	Patients for predicting lower limb function
**Demographic data**	
*Number of patients, n*	221
*Age, years*	65.0 ± 11.9
*Days to transfer or admission*	30.2 ± 72.2
**Clinical data**	
*Initial MBC*	2.4 ± 1.8
*Initial FAC*	1.0 ± 1.2
*Initial MRC*	
Shoulder abduction	1.5 ± 1.4
Elbow flexion	1.6 ± 1.5
Finger flexion	1.3 ± 1.5
Finger extension	1.1 ± 1.5
Hip flexion	2.0 ± 1.4
Knee extension	2.1 ± 1.5
Ankle dorsiflexion	1.5 ± 1.5

*MBC* modified Brunnstrom classification, *FAC* functional ambulation category, *MRC* medical research council.

### Images used for deep learning (input variables)

**Figure 2:**
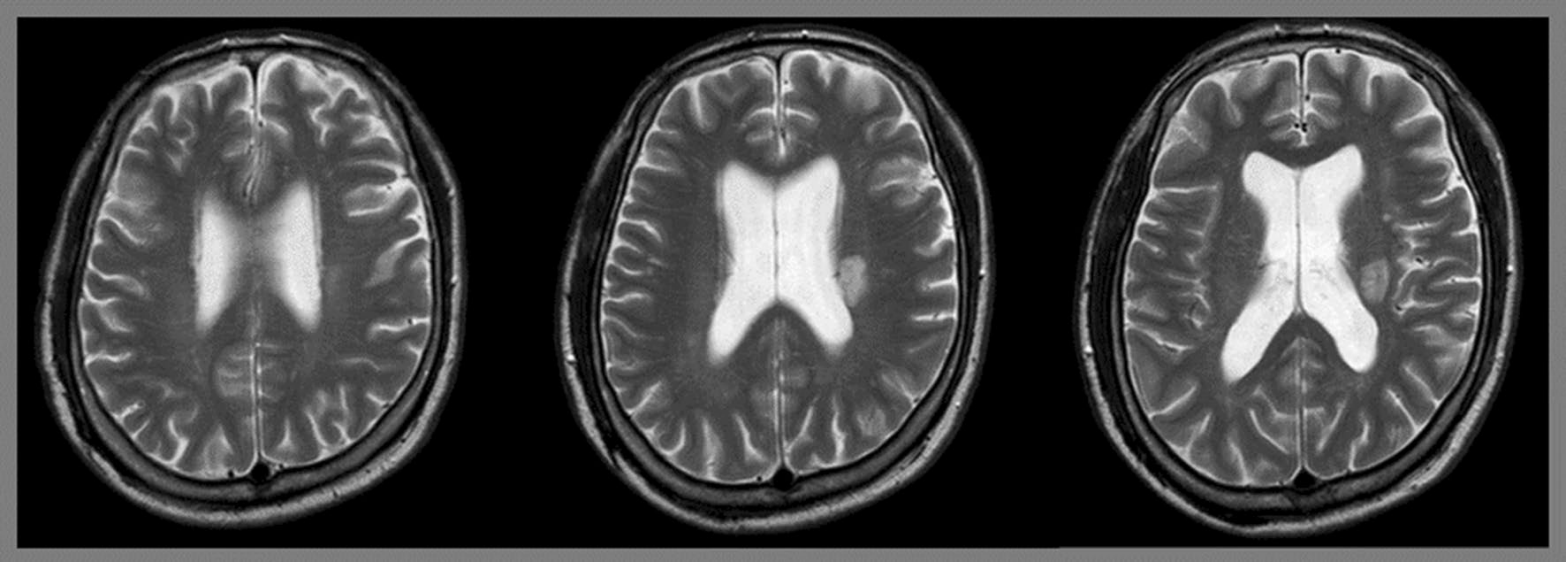
Three axial consecutive brain T2-magnetic resonance images from a 67-year-old man with left corona radiata infarct.

Three T2-axial consecutive brain MR images obtained from each patient were used in our study. The images at the levels of the body of the lateral ventricle were collected, such that corona radiata fibers passing above the internal capsule could be observed (Fig. 2). The MR images captured on the day closest to the date of transfer to the rehabilitation department, within 30 days after infarction onset, were used for the analysis.

### Motor outcome at six months (output variables)

We used the FAC score as the output data. The FAC is used for quantifying ambulatory function (walking ability), and it is based on the characterization of the levels of assistance required during a 15 m walk^19^. The FAC is categorized as follows: 0: nonambulatory, 1: continuous support from one person necessary, 2: intermittent support from one person necessary, 3: requirement of verbal supervision only, 4: assistance required on stairs and uneven surfaces, and 5: can walk independently anywhere. We categorized the FAC at six months after the onset as follows: favorable outcomes for lower limbs: FAC score ≥ 4 (able to walk without a guardian’s assistance) and poor outcomes for lower limbs: FAC score < 4.

### Deep learning algorithms

We used a CNN algorithm implemented using the Python programming language. Tensorflow 2.3, the Keras framework, and scikit-learn toolkit 0.23.2 were used to train the ML model. The details of the model and its performance are described in Table 2. The classifier with a majority voting algorithm was employed to predict the motor outcomes of individual patients. For example, when the predictions corresponding to two images were favorable outcomes and that corresponding to a third image was a poor outcome, the final decision for the patient was a favorable outcome.

**Table 2:** Performances of the deep-learning model.

ML model	FAC prediction model
Sample size (patients)	154 for training, 67 for validation, total 221
Sample size(images)	462 for training, 201 for validation, total 663
Sample zero ratio	Train 54.55%, validation 53.73%
CNN model details	- Mobilenet V1 with fine tuning Binary classification with sigmoid activation SGD(Stochastic Gradient Descent) optimizer, elu activation, batch size 32 Dropout regularization - Training accuracy: 73.59% - Validation accuracy: 71.64%
FAC classifier performance	- Validation accuracy: 76.12% - Validation AUC 0.751 with CI [0.649–0.852]

*ML* machine learning, *FAC* functional ambulation category, *DNN* deep neural network, *SGD* stochastic gradient descent, *AUC* area under the curve, *CI* confidence interval.

Among the included subjects, 69.7% (n = 154) were selected randomly for the training set and the remaining 30.3% (n = 67) were assigned to the validation set to measure the model performance.

### Statistical analysis

A receiver operating characteristic curve analysis was performed, and the area under the curve (AUC) was calculated using scikit-learn. The confidence interval for the AUC was calculated using the approach used by DeLong et al.^20^.

## Data Availability

Some or all data, models, or code generated or used during the study are available from the corresponding author by request.
